# Characterization of Developmental- and Stress-Mediated Expression of Cinnamoyl-CoA Reductase in Kenaf (*Hibiscus cannabinus* L.)

**DOI:** 10.1155/2014/601845

**Published:** 2014-02-26

**Authors:** Ritesh Ghosh, Bosung Choi, Byoung-Kwan Cho, Hyoun-Sub Lim, Sang-Un Park, Hyeun-Jong Bae, Savithiry Natarajan, Hanhong Bae

**Affiliations:** ^1^School of Biotechnology, Yeungnam University, Gyeongsan 712-749, Republic of Korea; ^2^Department of Biosystems Machinery Engineering, Chungnam National University, Daejeon 305-764, Republic of Korea; ^3^Department of Applied Biology, Chungnam National University, Daejeon 305-764, Republic of Korea; ^4^Department of Crop Science, Chungnam National University, Daejeon 305-754, Republic of Korea; ^5^Department of Bioenergy Science & Technology, Chonnam National University, Gwangju 500-757, Republic of Korea; ^6^US Department of Agriculture, Agricultural Research Service, Invasive Insects Biocontrol & Behavior Laboratory, 10300 Baltimore Avenue, Beltsville, MD 20705, USA

## Abstract

Cinnamoyl-CoA reductase (CCR) is an important enzyme for lignin biosynthesis as it catalyzes the first specific committed step in monolignol biosynthesis. We have cloned a full length coding sequence of *CCR* from kenaf (*Hibiscus cannabinus* L.), which contains a 1,020-bp open reading frame (ORF), encoding 339 amino acids of 37.37 kDa, with an isoelectric point (pI) of 6.27 (JX524276, *HcCCR2*). BLAST result found that it has high homology with other plant CCR orthologs. Multiple alignment with other plant CCR sequences showed that it contains two highly conserved motifs: NAD(P) binding domain (VTGAGGFIASWMVKLLLEKGY) at N-terminal and probable catalytic domain (NWYCYGK). According to phylogenetic analysis, it was closely related to CCR sequences of *Gossypium hirsutum* (ACQ59094) and *Populus trichocarpa* (CAC07424). *HcCCR2* showed ubiquitous expression in various kenaf tissues and the highest expression was detected in mature flower. *HcCCR2* was expressed differentially in response to various stresses, and the highest expression was observed by drought and NaCl treatments.

## 1. Introduction

**Figure 1:**
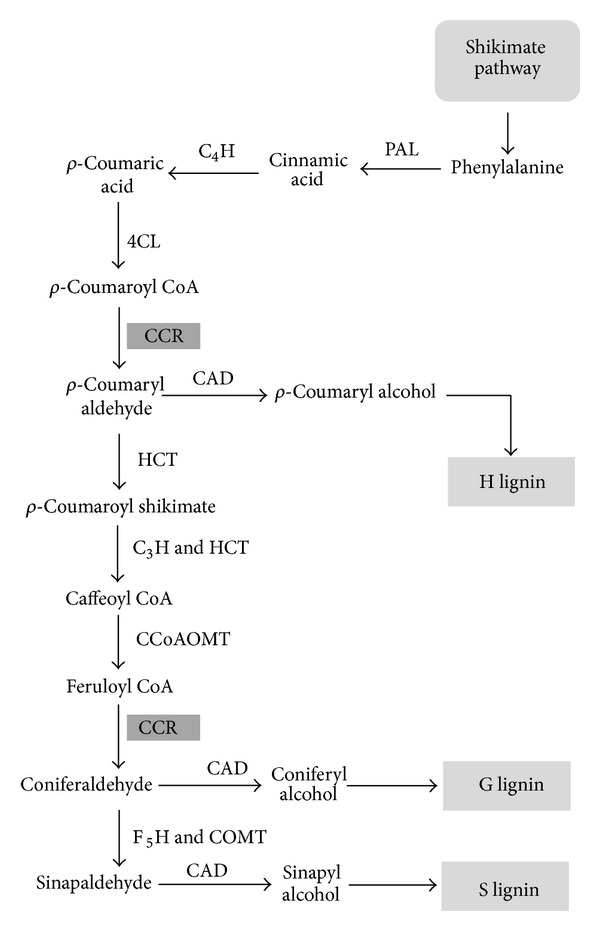
Monolignol biosynthetic pathway in dicotyledonous angiosperms. PAL, phenylalanine ammonia-lyase; C4H, cinnamate 4-hydroxylase; 4CL, 4-coumarate: CoA ligase; HCT, *p*-hydroxycinnamoyl-CoA: quinate shikimate *p*-hydroxycinnamoyltransferase; C3H, *p*-coumarate 3-hydroxylase; CCoAOMT, caffeoyl-CoA *O*-methyltransferase; CCR, cinnamoyl-CoA reductase; CAD, cinnamyl alcohol dehydrogenase; COMT, caffeic acid *O*-methyltransferase; F5H, ferulate 5-hydroxylase. Modified from Godfrey Neutelings (2011) [2].

Lignin is an aromatic heteropolymer and normally present in the secondary thickened plant cell walls with cellulose and hemicellulose [1–3]. It is the second most abundant biopolymer in the earth, after cellulose. It gives rigidity to the plant cell wall and confers hydrophobicity to vascular elements [4]. Besides providing mechanical support, it creates a strong barrier to pathogen invasion [4]. Lignification can be induced by pathogen attack, wounding, and other abiotic stresses [5]. It is believed that emergence of lignin during evolution is a crucial adaptation for plants to live on land [6]. In addition to vascular plants, some bryophytes and red algae also contain lignin or lignin-like molecules [3]. Lignin biosynthesis is very complex and involves several enzymes. It is produced by the phenylpropanoid pathway (Figure 1). There are two major steps of lignin biosynthesis in plants: monolignol biosynthesis (coniferyl alcohol, sinapyl alcohol, and *ρ*-coumaryl alcohol) and cross-linking of the monolignols. Cross-linking is conducted by peroxidases and laccases to form polymers [6]. Intercellular synthesis of three monolignol precursors is followed by extracellular transport and polymerization as guaiacyl (G), syringyl (S), and *ρ*-hydroxyphenyl (H) units of lignin, respectively [1–3]. In addition to these three monolignols, other phenylpropanoids also incorporate in the polymer [4]. Composition of monolignol units and amount of lignin are highly variable between taxa and cell types, even in different environmental conditions [2–4]. Lignin is problematic during pulp and biofuel production [7, 8]. The presence of lignin decreases forage digestibility to cattle and other ruminants. Recently, researches focus on the development of genetically modified plants with less lignin content or altered composition. In this context, cinnamoyl-CoA reductase (CCR) can be a good target gene. CCR catalyzes the first specific committed step in monolignol biosynthesis [9, Figure 1]. CCR enzyme converts different cinnamoyl-CoA esters (*ρ*-coumaroyl-CoA, caffeoyl-CoA, feruloyl-CoA, and sinapoyl-CoA) to corresponding cinnamaldehydes [10]. However, substrate specificity varies between different CCR enzymes from different species, even in between isoenzymes from the same species [11]. CCR orthologs were identified from various plants [12, 13]. Plants with downregulated* CCR *and* CCR*-like genes caused various phenotypic and developmental abnormalities: dwarfism, reduced number of seeds, small stem diameter, shorter floral stems, dark green leaves, growth delay, and collapsed xylem vessels [11–14]. Downregulation of CCR enzyme reduced lignin content in *Arabidopsis* and tobacco up to 50% [7]. In tomato, downregulation of CCR also decreased lignin content and increased soluble phenolic pools [15]. CCR gene family is very diverse and can be classified as true and CCR-like [11]. Multiple homologs of CCR genes can be present in the same plant and they are involved in different function. In case of *Arabidopsis*, there are 11 *CCR* homologs [11]. Among them *AtCCR1* is involved in developmental lignification, while *AtCCR2* is for stress and elicitor response [4].

Kenaf (*Hibiscus cannabinus *L.), an annual dicotyledonous plant, is a potential source for future biomass production [16]. It has high growth rate and broad ecological adaptability and can grow in adverse environmental condition [17, 18]. Kenaf bark (35–40% of total stem weight) is a good raw material for high quality of paper production. Its stem is composed of two types of fibers: outer long fiber (2–6 mm) and inner short fiber (0.6 mm) in 1 : 3 ratios [19]. Beside paper industry, kenaf stem is highly valuable for packaging materials, textiles, and bio-composite materials [19, 20]. In the present study, a *CCR* from kenaf was isolated and characterized based on sequence homology. Expression patterns in different tissues were investigated. Effects of various stresses and elicitors on *CCR* expression were also investigated. For this purpose, various stresses (MeJA (methyl jasmonic acid), cold, H_2_O_2_, SA (salicylic acid), ABA (abscisic acid), wounding, NaCl, and drought) were applied and the expression patterns were analyzed in 3-week-old stem tissues of kenaf plants.

## 2. Materials and Methods

### 2.1. Plant Materials, Stress Treatments, and RNA Isolation

Kenaf (*Hibiscus cannabinus *L., C-9) was grown and treated as described previously [21]. Three-week-old kenaf seedlings were treated with MeJA, cold, H_2_O_2_, SA, ABA, wounding, NaCl, and drought. RNA was extracted as described previously [21].

### 2.2. Cloning

Two micrograms of RNA were used for cDNA synthesis using Superscript III First-strand synthesis kit (Invitrogen, Carlsbad, CA, USA) according to the manufacturer's instructions. Gene specific primers were used to amplify from cDNA [CCR-F, 5′-AA(T/C)CC(A/T)GATGATCC-3′; CCR-R, 5′-TCCATGTA(C/G/A)AC(T/G/C)GCACC-3′]. The degenerate primers were designed based on the consensus sequences of the *CCR* orthologs of *Arabidopsis thaliana* (NM101463), *Raphanus raphanistrum *(EV527773), *Glycine max* (AK286730), *Betula luminifera *(FJ410450), *Capsicum annuum* (EU616555), and *Brassica rapa* (EX046473). The PCR product was confirmed by running in a 1.2% agarose gel and then it was purified using Wizard SV Gel and PCR Clean-up System (Promega, Madison, WI, USA) and cloned into pGEM-T easy Vector (Promega). DNA sequences were analyzed by Cosmogenetech Co. (Seoul, Korea). For cloning of full length *CCR* ortholog, both 5′ and 3′ RACE (rapid amplification of cDNA ends) were performed using the RACE systems according to the manufacturer's instructions (Invitrogen).

### 2.3. Quantitative Real-Time PCR (QPCR) Analysis

QPCR was performed as described previously [22]. Mx3000P QPCR System (Agilent, Santa Clara, CA, USA) with SYBR Green QPCR Master Mix (LPS Solution, Daejeon, Korea) were used. Primers were designed using Primer 3 software of Biology Workbench (http://workbench.sdsc.edu/). Forward and reverse primers of *HcCCR2* ortholog were as follows: forward primer, 5′-AAGTTCTCGAACCAGAAGCTG AG-3′; reverse primer, 5′-TGCGTCTCCACTTCCCTTAATAAACC-3′. *ACTIN* (DQ866836), a housekeeping gene, was used as an expression control with the primer sequences: forward primer, 5′-AAGTTCTCGAACGAGAAGCTGAT-3′; reverse primer, 5′-AGTGATTTCCTTGCTCATACGGT-3′.

### 2.4. Data Analyses

DNA and protein sequences were analyzed using NCBI Blast (http://blast.ncbi.nlm.nih.gov/), Biology WorkBench (ClustalW), ExPASy Proteomics Server (http://expasy.org/tools/pi_tool.html), Superfamily 1.75 (http://supfam.org/SUPERFAMILY/index.html/), SignalP 3.0 (http://www.cbs.dtu.dk/services/SignalP/), and TargetP V1.1 (http://www.cbs.dtu.dk/services/TargetP/). Phylogenetic tree was constructed using amino acid sequences by the neighbor joining method in Mega5 (http://www.megasoftware.net/).

## 3. Results and Discussion

### 3.1. Cloning and Sequence Analysis

**Figure 2:**
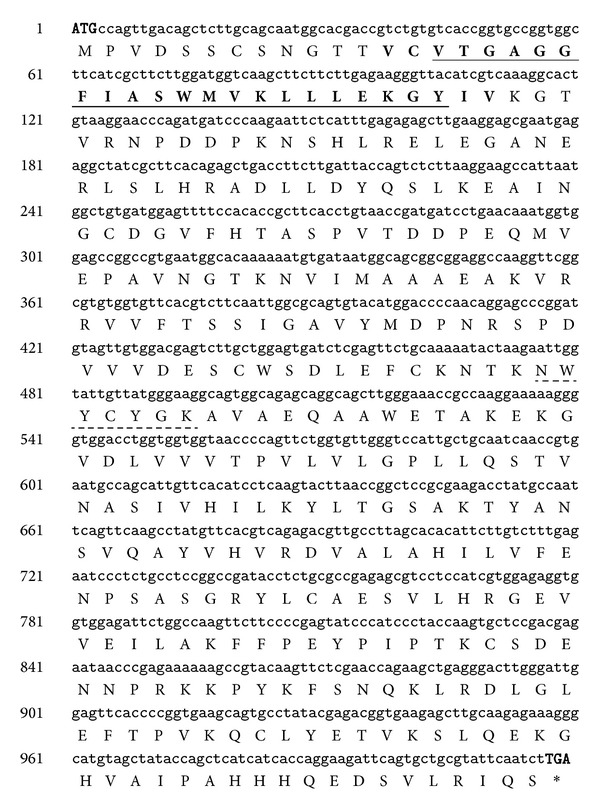
Full length CDS and deduced amino acid sequence of kenaf CCR1 ortholog. The start codon (ATG) and stop codon (TGA) are in uppercase. Putative NAD(P) binding domain and catalytic domain are underlined in solid and dashed line, respectively.

**Figure 3:**
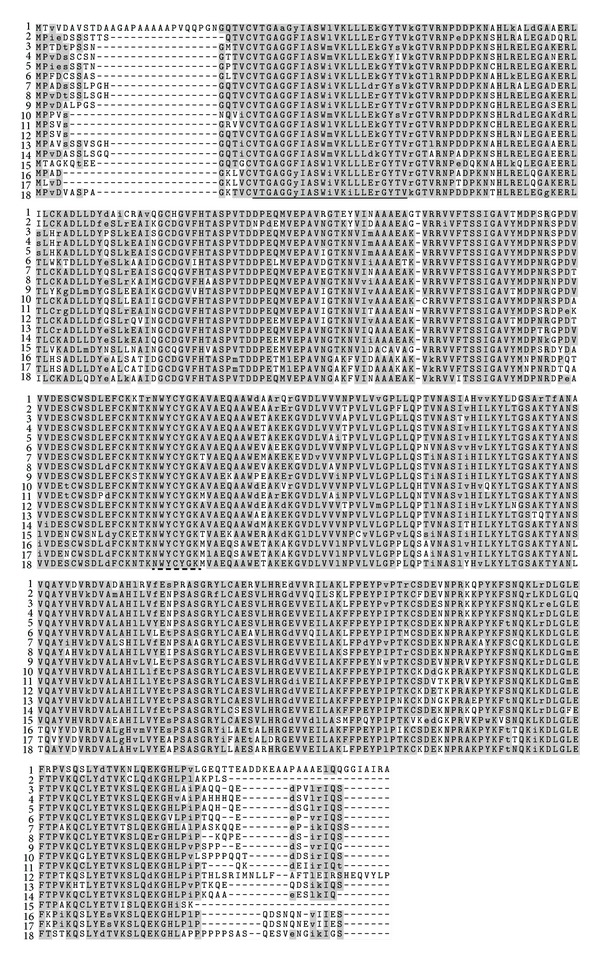
Multiple alignment of the deduced amino acid sequences of kenaf CCR ortholog with other plants by using ClustalW and BOXSHADE sequence alignment program in Biology WorkBench. Identical and similar amino acids are highlighted with gray. Conserved NAD(P) binding domain and catalytic domain are underlined in solid and dashed line, respectively. GenBank accession numbers are represented as follows: (1) *Saccharum officinarum *(CAA13176), (2) *Cinnamomum osmophloeum *(AFG26325), (3) *Hibiscus cannabinus *(ADK24219), (4) *Hibiscus cannabinus *(JX524276), (5) *Gossypium hirsutum *(ACQ59094), (6) *Betula luminifera *(ACJ38670), (7) *Linum album *(CAD29427), (8) *Hevea brasiliensis *(ADU64758), (9) *Eucalyptus gunnii *(CAA56103), (10) *Codonopsis lanceolata *(BAE48787), (11) *Solanum lycopersicum *(NP001234612), (12) *Vaccinium corymbosum *(ACI14382), (13) *Pyrus pyrifolia *(ADK62523), (14) *Populus trichocarpa *(CAC07424), (15) *Pinus massoniana *(ACE76870), (16) *Brassica napus *(AEK27156), (17) *Arabidopsis thaliana *(AAG53687), and (18) *Arabidopsis thaliana *(NP173047).

**Figure 4:**
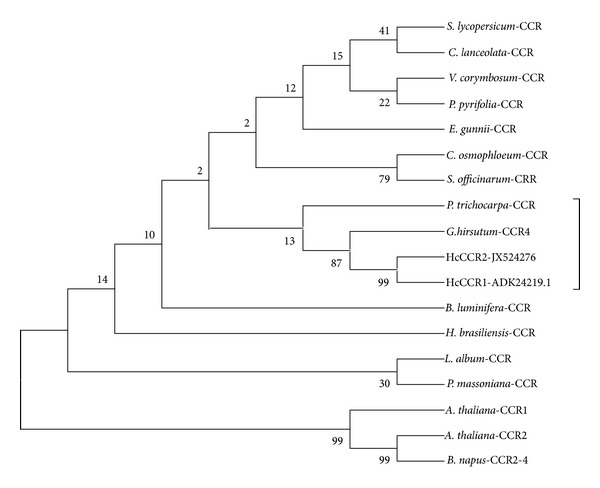
Phylogenetic analysis of the deduced amino acid sequences of kenaf CCR ortholog and other plants. The tree was constructed by the neighbor-joining method of ClustalW and Mega5. The numbers at the nodes indicate bootstrap values from 1000 replications. GenBank accession numbers are represented as follows: *Solanum lycopersicum *(NP001234612), *Codonopsis lanceolata *(BAE48787), *Vaccinium corymbosum *(ACI14382), *Pyrus pyrifolia *(ADK62523), *Eucalyptus gunnii *(CAA56103), *Cinnamomum osmophloeum *(AFG26325), *Saccharum officinarum *(CAA13176), *Populus trichocarpa *(CAC07424), *Gossypium hirsutum *(ACQ59094), *Hibiscus cannabinus *(JX524276), *Hibiscus cannabinus *(ADK24219),* Betula luminifera *(ACJ38670), *Hevea brasiliensis *(ADU64758), *Linum album *(CAD29427),* Pinus massoniana *(ACE76870), *Arabidopsis thaliana *(NP173047),* Arabidopsis thaliana *(AAG53687), and *Brassica napus *(AEK27156).

We cloned a full length of *CCR *ortholog from kenaf (GenBank Accession number JX524276). For full length cloning, we used degenerate primers and RACE system. Sequencing data suggested that it consists of a 1,020-bp open reading frame (ORF), encoding 339 amino acids (Figure 2). The predicted molecular weight of the deduced protein is 37.37 kDa, with an isoelectric point (pI) of 6.27, as calculated by the ExPASy Proteomics Server. BLASTP search reveled that deduced protein sequence has high similarities with other plant CCR sequences. Among them, one is another CCR ortholog from kenaf (ADK24219). According to Target P analysis, JX524276 proteins have no signals for subcellular localization, which suggests that our CCR protein is probably cytoplasmic. Previous study also showed that OsCCR1-GFP localized to the cytoplasm when it was transiently expressed in rice protoplast [23]. Signal P 3.0 analysis also showed no significant signal peptides at N terminal which suggests that JX524276 probably codes a nonsecretory protein. Multiple alignments of CCR protein sequences showed high sequence identities with other CCRs, up to 73% (Figure 3). Among them *Gossypium hirsutum* (ACQ59094) is the highest with 90% identity. The alignment result also showed two highly conserved motifs: NAD(P)-binding domain at N-terminal (VTGAGGFIASWMVKLLLEKGY) and probable catalytic domain (NWYCYGK) [12, 13]. Consensus sequence for NAD(P) binding domain is VTGA(G/A)G(F/Y)(I/L)ASW(I/L/M)VK(L/I)LL(E/D)(K/R)GY. Putative catalytic domain (NWYCYGK) is fully conserved among the species. Superfamily result also predicted that this kenaf CCR belongs to NAD(P) binding Rossmann-fold domain containing protein. Previous literature suggested that all plant CCRs can be broadly classified in two groups: CCR and CCR-like [4, 11]. It is predicted that only one or two true *CCR* genes are present in plant for lignin biosynthesis during development, whereas others are backup for the real one [11]. As an example, experimental evidence showed that *Arabidopsis* has only two real *CCR* genes out of eleven *CCR* homologs [9]. *AtCCR2* expression was increased in *Arabidopsis ccr1* mutant and function was partly compensated [14]. CCR enzyme converts different cinnamoyl-CoA esters, such as *ρ*-coumaroyl-CoA, caffeoyl-CoA, feruloyl-CoA, 5-hydroxyferuloyl-CoA, and sinapoyl-CoA, to corresponding cinnamaldehydes [10]. However, substrate specificity varies between different CCR enzymes from different species, even in between isoenzymes from the same species [11]. In order to study the evolutionary relationships among different CCR sequences from various plants, a phylogenetic tree was constructed (Figure 4). Among 18 members of plant CCR proteins, both of kenaf CCR orthologs showed the closest relationship to *Gossypium hirsutum* (ACQ59094) and *Populus trichocarpa* (CAC07424). These results indicate that we successfully cloned a *CCR* ortholog from kenaf. From now on, ADK24219 and JX524276 are designated as *HcCCR1* and *HcCCR2*, respectively.

### 3.2. Analysis of Tissue Specific Expression of *HcCCR2*

**Figure 5:**
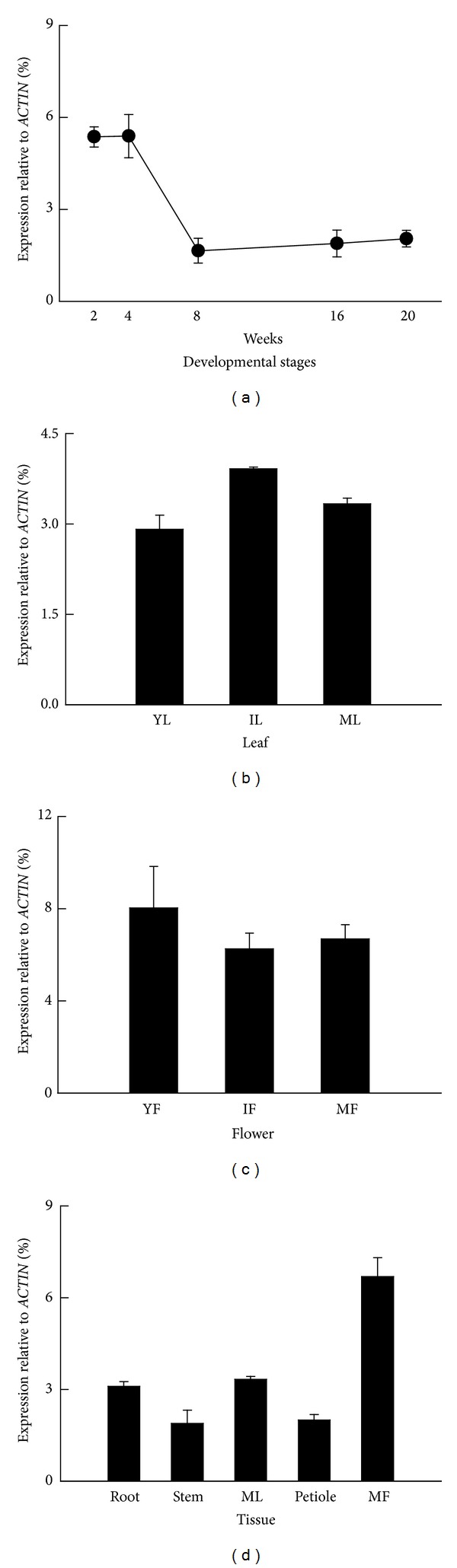
Transcript expression patterns of *HcCCR2* in various tissues and organs during developmental stages. Relative transcript levels were measured using QPCR and *ACTIN* transcript were used as internal control. The transcript levels of kenaf *CCR* ortholog were adjusted after deduction of the control transcript level: (a) during stem development (2, 4, 8, 16, and 20 weeks after sowing), (b) during leaf development (YL, young leaf; IL, immature leaf; ML, mature leaf), (c) during flower development (YF, young flower; IF, immature flower; MF, mature flower), and (d) expression pattern in various tissues and organs from 16-week-old kenaf plants. Bars show means ± standard error of 3 biological replications.

We investigated the expression patterns of *HcCCR2 *transcripts in different tissues (Figure 5). During stem development, *HcCCR2 *was highly expressed up to 4 weeks. Then it was sharply downregulated in 8-week-old plants and maintained its steady state up to 20 weeks (Figure 5(a)). *HcCCR2 *did not show big differential expression among different leaf tissues, though immature leaf showed higher expression compared to young and mature leaves (Figure 5(b)). In flower, *HcCCR2 *showed higher level of expression in young flower, compared to immature and mature flower tissues (Figure 5(c)). *HcCCR2* transcripts were identified in all tissues and organs of 16-week-old plants (Figure 5(d)). The expression pattern of *HcCCR2* can be divided into three classes: (1) high expression in mature flower; (2) intermediate expression in root and mature leaf; (3) low expression in stem and petiole. Higher expression at mature flower suggests that *HcCCR2* might have an important role during flowering. Expression of the genes involved in lignin biosynthesis is important for fertility (pollen release) and seed dispersal (silique dehiscence) [24]. High levels of phenylpropanoid-derived compounds were also detected in *Arabidopsis* flowers [25]. Other phenylpropanoid pathway related genes (*C3H*, *HCT*, *CCoAOMT*, *PAL*, and* C4H*) also showed high expression in flower tissues of kenaf plants [21, 26–29]. CCR has important role in development.* Arabidopsis* CCR1 has conserved AC elements in promoter, which are responsible for developmental lignification [4]. Previous studies showed that *CCR* family has diverse expression patterns among genus, species, and even in different tissues of the same plant. In *Arabidopsis*, *AtCCR1* was highly expressed in all tissues compared to *AtCCR2* [4]. Ten poplar *CCR* homologs differentially expressed in bark, leaf, and xylem tissues [11]. Among them *CCR2 *and* CCR6* showed highest expression in leaf tissues. Some *CCR* genes from *Isatis, Ginkgo,* and Norway spruce highly expressed not only in lignified tissues like stems, but also in other tissues [13, 30, 31]. Those results suggest that different *CCR* homologs might be involved in different cellular function.

### 3.3. Analysis of Stress-Regulated Expression of *HcCCR2* in Stem Tissues

**Figure 6:**
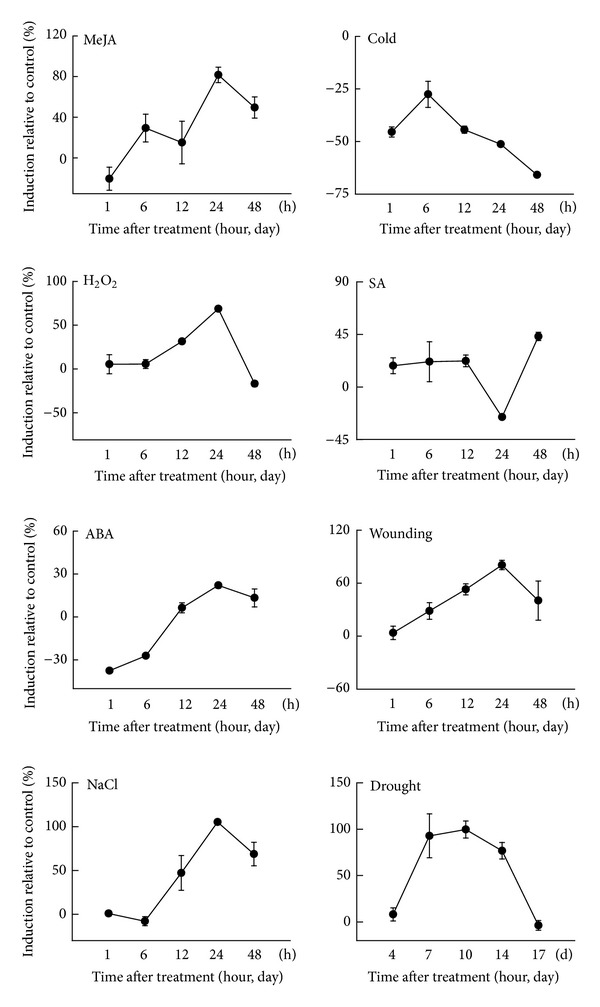
Transcript expression patterns of *HcCCR2* in response to various abiotic stresses. Three-week-old stem tissues were treated with different stresses such as wounding, salicylic acid (SA), NaCl, cold, H_2_O_2_, methyl jasmonate (MeJA), abscisic acid (ABA), and drought. Bars show means ± standard error of 3 biological replications.

As plants are sessile, they are facing many adverse environmental conditions throughout their life span. Plants have developed advanced mechanisms to defend themselves from various biotic and abiotic stresses. Lignification is one of the most important mechanisms to combat with stresses [5]. Not much has been known about the stress-mediated *CCR* expression in plants. Various stresses were applied to 3-week-old kenaf plants in order to examine the expression patterns of *HcCCR2* transcripts in stem tissues (Figure 6). All treatments showed differential expression of *HcCCR2*. Wound, NaCl, ABA, and H_2_O_2_ treated samples showed gradual upregulation up to 24 h, and then the expression was decreased at 48 h. Maximum expression was occurred at 24 h by all these four treatments. MeJA treated samples also showed highest expression at 24 h. SA treated samples did not show differential expression up to 12 h. Then it was downregulated at 24 h, followed by upregulation at 48 h. Cold treated samples showed quite different expression pattern. In cold treated samples *HcCCR2* was gradually downregulated except 6 h. Drought induced the expression up to 10 days, then it was downregulated. Among the treatments, highest accumulation of *HcCCR2* was observed in NaCl (% relative expression to *ACTIN*) at 24 h and drought treatment (% relative expression to *ACTIN*) at 10 days. These results suggest that *HcCCR2* is involved in stress regulatory pathway. Previous literature also showed stress mediated differential expression of *CCR* in various plant species. Water deficit treatments induced the expression of *ZmCCR1 *and* ZmCCR2* as well as lignin biosynthesis in maize root elongation zone [32]. This upregulation was detected both at 1 and 48 h after water deficit treatment. Particularly in 48 h *ZmCCR2* showed 10fold upregulation compared to control. MeJA treatment showed high induction of *IiCCR *gene from *Isatis indigotica*, especially at 4 and 8 h after treatment [13]. ABA treated plant showed downregulation of *IiCCR* transcript, which is similar to our cold treated sample. Other groups showed ABA induced *GmCCR* transcript in soybean [33]. SA treatment also induced *CCR *transcripts in *Arabidopsis* leaves and *Linum album *cell cultures [34]. They also showed wounding and NaCl mediated upregulation of *GmCCR *transcript. Probably different homologs are involved in different function.* CCR* is also responsive to various biotic stresses. For example, fungal and bacterial infection induced *CCR* genes: wheat *TmCCR* by powdery mildew; switchgrass *PvCCR2* by *Puccinia*; *Arabidopsis AtCCR2* by *Xanthomonas campestris* pv. *Campestris *[35–37]. It is hypothesized that OsRac1, one of the Rac/Rop family of small GTPases, activates CCR activities upon pathogen attack, which results in the induction of monolignol production [23]. We can divide all treatments into two categories, though they are interlinked with each other. NaCl, drought, cold, and ABA exert similar kind of stress on the plant [38–40]. In other hand SA, JA, and wounding are related to the pathogen mediated signaling pathway [41]. Mechanical wounding and pathogen cause similar responses in plant. Both of them induce cellular phytohormone (SA, JA, and ABA) and lignification to the damaged or invading sites [41, 42]. These phytohormones help to propagate and amplify the perceived signal via both synergistic and antagonistic interactions [41, 43]. H_2_O_2_ is a very important signaling molecule for all kind of stresses. H_2_O_2_ and other reactive oxygen species (ROS) can be produced by different stresses, which in turn cause random cross-linking of subunits and formation of lignin [44]. Previous experiment showed that many lignin biosynthesis genes were differentially expressed due to the various stress treatments. Cold induced phenylalanine ammonia lyase (PAL) activity in *Brassica napus*, cinnamate 3-hydroxylase (C3H) expression in *Rhododendron*, and *HcCCoAOMT *and* HcHCT *expression in kenaf [5, 21, 27] were reported.

In summary, we have cloned the full length coding sequence of cinnamoyl-CoA reductase (*HcCCR2*) from kenaf, which is probably homologous with the previously reported kenaf *HcCCR1*. *HcCCR2* ubiquitously expressed in different tissues and showed differential expression in response to various stress treatments in different amplitude. According to our knowledge, in this paper, for the first time we have characterized expression pattern of kenaf *CCR* in different tissues and under various stress treatments, though further comparative investigation between two kenaf CCR homologs are required to know the substrate specificity and involvement in developmental lignification and stress tolerance.
